# On Tinea Capitis

**Published:** 1817-02

**Authors:** 


*For the London Medical and Physical Journal.*

### *On Tinea Capitis;* by L. G.

IT is said by a celebrated teacher in one of our schools of surgery, that there is no sure remedy for tinea capitis. —This saying is probably true; at the same time, a certain degree

---

degree of benefit may, perhaps, be reasonably expected from all the means in common use. It is my present object to add to the list of these means, premising that none of those who may read this account can be more sceptical as to the success of the remedy, upon a further trial, than I am myself; knowing, as I do, that the best medical experience furnishes an evidence so imperfect, that we are more or less deceived by it every day of our lives.

I was induced to employ turpentine, between two and three years since, in an inveterate case of tinea capitis; not because half the world are turpentine-mad, for, among all my vices, I am not celebrated as a copyist; but, from a theory, or perhaps half a dozen theories, of my own, trumpery ones most likely, and therefore not worth mentioning. The disease got well under the use of turpentine, in less than three weeks. Since that time, I have employed it in eight or ten cases, and in all with the same success; that is, the disease has got well in two, three, or four weeks.—So much for the grounds of its recommendation. The plan adopted in the use of it is as follows:—A liniment to be composed of Sp. Terebinth. ℥i. Ol. Oliv. ℨij. The scalp (kept regularly shaved) is to be well washed every morning with soft soap and hot water; it is then to be wiped dry, and the liniment to be rubbed from five to ten minutes, upon all the places affected by the disease. On the following morning, the ablution, and then the friction, &c. are to be repeated. A clean cap should be put on every day. After the liniment has been used a few days, a degree of inflammation is excited, which is sometimes considerable, perhaps accompanied with erythema, the scrabs separate, &c. The plan is to be persevered in, and the inflammation kept up until the disease ceases. I have also, in conjunction with the above plan, given a powder with calomel and jalap, perhaps four doses in the course of a fortnight. In one case of near two years standing, the inflammation of the scalp was very violent, extending behind the ear down the neck, in which latter part a gland suppurated, burst, and soon got well. Whether this was the effect of the turpentine, I cannot say, but I have met with it in no other instance. The disease, in this case, extended nearly over the whole scalp, and it was well in about a fortnight from the commencement of the above plan. I have three children now under my care, with whom the same treatment is adopted, and apparently with the same benefit. The strength of the liniment may be varied, and its use either suspended, or its repetition may be more frequent, according to its visible effects, and the discretion of the practitioner. My theory requires that a certain a certain degree of inflammation should be unremittingly maintained; this may be necessary, and yet the theory may be false, as theories are apt to be.

My object in troubling you with this communication is, that the merit of the means suggested may be ascertained: to do this satisfactorily, will require a more ample experience than falls to the lot of an individual. I am diffident of anticipating future from past success; eight or ten chronic disorders of the head have ceased under the use of purgatives; I have, in consequence, felt a confidence in such means; the same treatment has, however, failed in perhaps the next five cases, which were, in their symptoms, similar to those in which it appeared to succeed. The history of medicine furnishes many examples of an astonishing success of remedies in the hands of their projectors: with others they have failed in the very first cases, and their credit has never been restored. Fortune makes our sagacity her sport: and, as if our little faculties were not of themselves sufficiently prone to error, maliciously arranges her coincidences: she abuses us even on the strong ground of experience, and, by a long train of similar circumstances, deludes us into the gross error of mistaking accidental for regular phenomena.

*December* 5, 1816.

